# Non-nucleoside reverse transcriptase inhibitors: a review on pharmacokinetics, pharmacodynamics, safety and tolerability

**DOI:** 10.7448/IAS.16.1.18567

**Published:** 2013-09-04

**Authors:** Iris Usach, Virginia Melis, José-Esteban Peris

**Affiliations:** Department of Pharmacy and Pharmaceutical Technology, Burjassot, Valencia, Spain

**Keywords:** human immunodeficiency virus, non-nucleoside reverse transcriptase inhibitors, nevirapine, delavirdine, efavirenz, etravirine, rilpivirine, next-generation non-nucleoside reverse transcriptase inhibitors

## Abstract

**Introduction:**

Human immunodeficiency virus (HIV) type-1 non-nucleoside and nucleoside reverse transcriptase inhibitors (NNRTIs) are key drugs of highly active antiretroviral therapy (HAART) in the clinical management of acquired immune deficiency syndrome (AIDS)/HIV infection.

**Discussion:**

First-generation NNRTIs, nevirapine (NVP), delavirdine (DLV) and efavirenz (EFV) are drugs with a low genetic barrier and poor resistance profile, which has led to the development of new generations of NNRTIs. Second-generation NNRTIs, etravirine (ETR) and rilpivirine (RPV) have been approved by the Food and Drug Administration and European Union, and the next generation of drugs is currently being clinically developed. This review describes recent clinical data, pharmacokinetics, metabolism, pharmacodynamics, safety and tolerability of commercialized NNRTIs, including the effects of sex, race and age differences on pharmacokinetics and safety. Moreover, it summarizes the characteristics of next-generation NNRTIs: lersivirine, GSK 2248761, RDEA806, BILR 355 BS, calanolide A, MK-4965, MK-1439 and MK-6186.

**Conclusions:**

This review presents a wide description of NNRTIs, providing useful information for researchers interested in this field, both in clinical use and in research.

## Introduction

Infections with the human immunodeficiency virus (HIV) are typically treated with drug combinations consisting of at least three different antiretroviral drugs. Essential components of this highly active antiretroviral therapy (HAART) are HIV protease inhibitors (PIs), non-nucleoside and nucleoside reverse transcriptase inhibitors (NNRTIs and NRTIs), fusion inhibitors (FIs), CCR5 antagonists and integrase strand transfer inhibitors (INSTIs). Currently, preferred regimens use combinations of two NRTIs and either an NNRTI, a ritonavir-boosted PI or an INSTI, which have all resulted in decreased HIV RNA levels (<50 copies/mL) at 48 weeks and CD4 cell increases in the vast majority of patients [1]. Many of these regimens have comparable efficacy but vary to some degree in dosing frequency, pill burden, drug interactions and potential side effects. The choice of a regimen for a given individual is based on expected side effects, convenience, comorbidities, interactions with concomitant medications and genotypic drug-resistance testing [1].

Recent data suggest that virologic failure on first-line regimens mostly occurs due to either pre-existing (transmitted) drug resistance or suboptimal adherence [1]. Therefore, genotypic resistance testing and adherence to the treatment are fundamental criteria when selecting the most optimal initial antiretroviral regimen. Although the prevalence of NNRTI resistance is higher than PI resistance in antiretroviral naïve patients, it has been reported that patients receiving NNRTIs show a higher rate of adherence than patients receiving PIs [2]. Furthermore, some authors do not recommend the widespread use of PI-based first-line therapy, in spite of more favourable resistance implications of PI- versus NNRTI-based first-line therapy, due to resource limitations in some countries and lack of second-line regimens (for patients with failure of an initial antiretroviral therapy) based on other antiretroviral classes [3]. A disadvantage of PI-based regimens is the large number of drug-drug interactions, which may make their use in patients taking other medications more difficult. Regimens containing raltegravir, the most commonly used INSTI, have fewer drug-drug interactions than PI-based regimens. However, raltegravir, like NNRTIs, has a low genetic barrier to resistance, with the additional disadvantage (related to treatment adherence) of requiring twice-daily dosing. A newer INSTI, elvitegravir, can be administrated once daily combined with cobicistat (an inhibitor of elvitegravir metabolism) and two NRTIs [1].

NNRTIs are usually not recommended as components of second-line regimens because of an increased risk of resistance-related failures [3, 4].

There are two types of HIV, HIV-1 and HIV-2, and both can cause acquired immune deficiency syndrome (AIDS). Most AIDS infections are due to HIV type 1 (HIV-1) strains, while HIV-2 represents a significant minority of all HIV infections in some countries, such as Guinea-Bissau and Portugal [5]. There are important differences between HIV-1 and HIV-2 that provide insights into virus evolution, tropism and pathogenesis. In particular, HIV-2 is less readily transmitted and is generally less pathogenic than HIV-1 [6].

**Figure 1:**
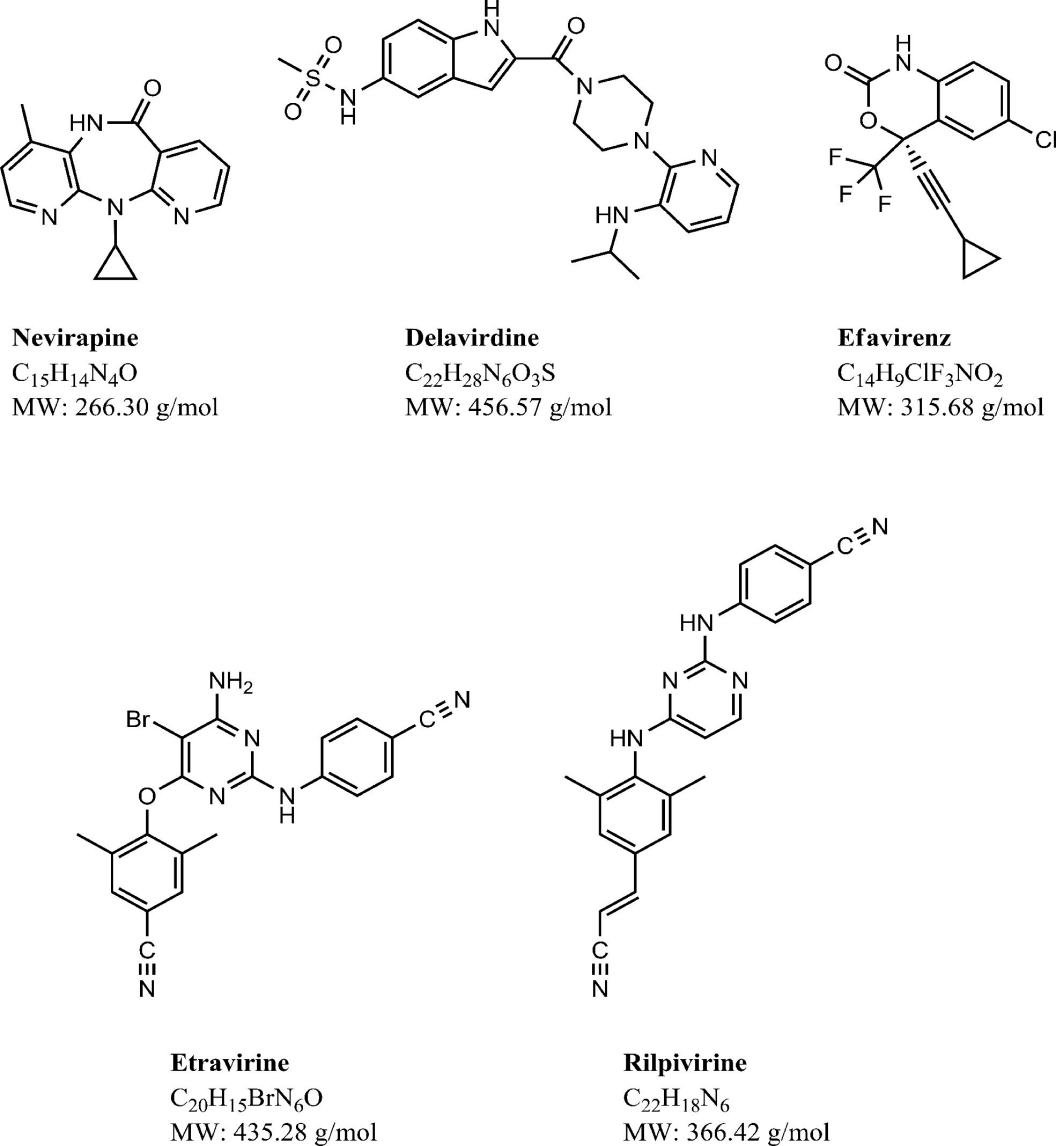
Chemical structure, molecular formula and molecular weight of first- and second-generation NNRTIs.

Five NNRTIs (nevirapine, NVP; delavirdine, DLV; efavirenz, EFV; etravirine, ETR; and rilpivirine, RPV) are currently US Food and Drug Administration (FDA) approved. Moreover, all of them except for DLV have been approved by the European Union. Their chemical structures and main characteristics are described in Figure 1 and Table 1, respectively. To improve adherence and reduce the risk of treatment errors, EFV (Atripla^®^) and RPV (Complera^®^/Eviplera^®^) have been co-formulated with two NRTIs (emtricitabine and tenofovir) as a single-tablet, once-daily regimen (Table 2). Atripla^®^ and Complera^®^/ Eviplera^®^ are recommended for treatment of HIV-1 infection when the agents included in the co-formulation are drugs of choice. However, they are not recommended in patients with creatinine clearance lower than 50 mL/min. In these patients, it is recommended to use the individual drugs of the fixed-dose combination and adjust tenofovir and emtricitabine doses according to creatinine clearance [1].

**Table 1:** Characteristics of NNRTIs approved by the European Union (EU) and/or the US Food and Drug Administration (FDA)

	Generic name (abbreviation)	Trade name	Manufacturer	Formulation	Date of FDA approval	Date of EU approval
	Nevirapine (NVP)	Viramune^®^ Viramune XR ^®^ (extended release)	Boehringer Ingelheim	• 200 mg tablet • Oral suspension (50 mg/5 mL) • 400 mg XR tablet	21 Jun. 1996 XR tablet: 25 Mar. 2011	5 Feb. 1998 XR tablet: 21 Sept. 2011
**First-generation NNRTIs**	Delavirdine (DLV)	Rescriptor^®^	Pfizer	100 and 200 mg tablets	4 Apr. 1997	–
	Efavirenz (EFV)	Sustiva^®^ (FDA and EU) Stocrin^®^ (EU)	Bristol-Myers Squibb Merck Sharp and Dohme Ltd	• 50, 100 and 200 mg capsules • 50, 200 and 600 mg tablets • Oral solution (30 mg/mL)	17 Sept. 1998	28 May 1999
**Second-generation**	Etravirine (ETR)	Intelence^®^	Janssen-Cilag	100 and 200 mg tablets	18 Jan. 2008	28 Aug. 2008
** NNRTIs**	Rilpivirine (RPV)	Edurant^®^	Janssen-Cilag	25 mg tablet	20 May 2011	28 Nov. 2011

**Table 2:** NNRTIs co-formulated with NRTIs in a single tablet and approved by the European Union (EU) and the US Food and Drug Administration (FDA)

Generic name	Trade name	Manufacturer	Formulation	Date of FDA approval	Date of EU approval
EFV with emtricitabine +tenofovir	Atripla^®^	Bristol-Myers Squibb and Gilead Sciences	(EFV 600 mg+emtricitabine 200 mg+tenofovir 300 mg^a^) tablet	12 Jul. 2006	13 Dec. 2007
RPV with emtricitabine+tenofovir	Complera^®^ (FDA) Eviplera^®^ (EU)	Gilead Sciences	(RPV 25 mg+emtricitabine 200 mg+tenofovir 300 mg^a^) tablet	10 Aug. 2011	28 Nov. 2011

a Expressed as tenofovir disoproxil fumarate.

All of these drugs prevent HIV-1 replication by non-competitively inhibiting reverse transcriptase (RT). This group is not active against HIV-1 strains in group O, HIV-2 or animal retroviruses. HIV-1 group O viruses are usually not encountered outside West and Central Africa; they are reportedly most common in Cameroon [7]. Each of the NNRTIs is metabolized to some degree by the cytochrome P450 (CYP) system of enzymes, mainly by CYP3A4, and glucuronoconjugation [8]. In addition, they elicit variable effects on other medications, acting as either inducers or inhibitors of drugs metabolized by CYP.

First-generation NNRTIs are drugs with a low genetic barrier that require only a single mutation to confer resistance, and cross-resistance affecting these NNRTIs is common. Instead, second-generation NNRTIs are compounds with a higher genetic barrier to resistance. All of them are generally safe and well tolerated, although hepatotoxicity and severe rash are associated with the use of NVP [9], whereas EFV causes central nervous system (CNS) side effects [10]. Most of them have a long plasmatic half-life (except DLV) and are given as a once-daily regimen.

**Figure 2:**
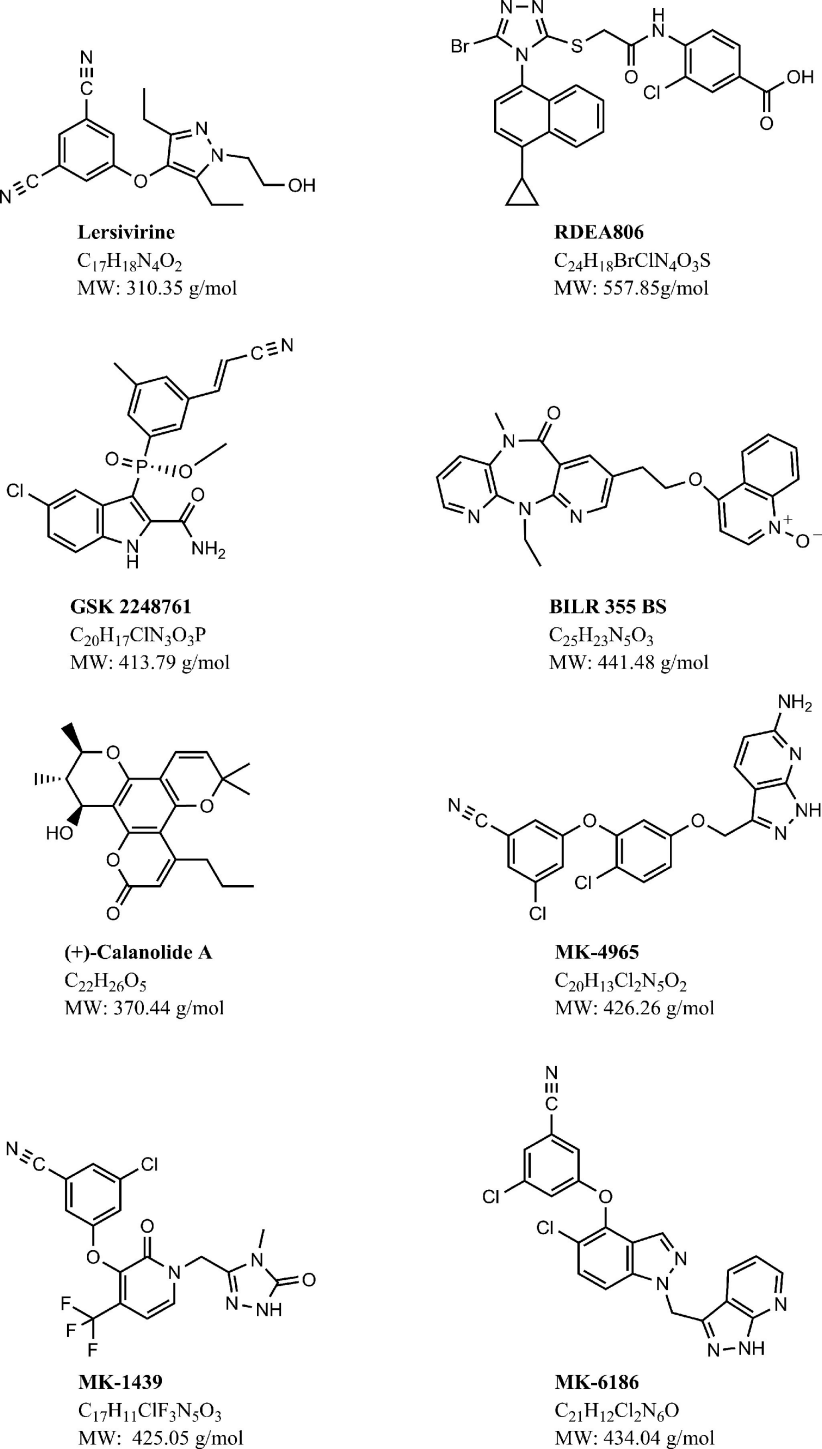
Chemical structure, molecular formula and molecular weight of next-generation NNRTIs.

At present, the next generation of NNRTIs is undergoing clinical development (Figure 2). The need for new therapeutic agents that are able to overcome resistance and safety problems prompted the development of new NNRTIs. These compounds are endowed with activity against wild-type HIV-1 and clinically relevant mutant strains. It is difficult for the new NNRTIs to prove their superiority with respect to the first- and second-generation NNRTIs, since therapy failure becomes rarer and rarer with the current antiretroviral combinations containing marketed NNRTIs.

The present article reviews the main characteristics of the different NNRTI drugs. It describes their pharmacokinetics and metabolism, pharmacodynamics, safety and tolerability.

## Discussion

### First-generation NNRTIs

The era of NNRTIs started with the discovery of 1-(2-2-hydroxyethoxymethyl)-6-(phenylthio)thymine (HEPT) [11] and tetrahydroimidazo[4,5,1-jkj][1,4]benzodiazepin-2(1H)-one and -thione (TIBO) [12] as specific HIV-1 inhibitors. Both HEPT and TIBO are highly active compounds against HIV-1, but they are inactive against HIV-2 or any other retrovirus, interacting with an allosteric site of HIV-1 RT.

Following HEPT and TIBO, several other compounds were identified as NNRTIs, including dipyridodiazepinones (i.e., NVP), bis(heteroaryl)piperazines (i.e., DLV) and benzoxazinones (i.e., EFV). These first-generation NNRTIs are approved by FDA for the treatment of HIV-1 infection, and their pharmacokinetic characteristics are summarized in Table 3 [13–23].

**Table 3:** Pharmacokinetic parameters of first- and second-generation NNRTIs [13–23]

	First-generation NNRTIs				Second-generation NNRTIs	
		
	NVP	NVP (extended release)	DLV	EFV	ETR	RPV
Dosage (mg)	200, bid	400, opd	400, tid	600, opd	200, bid	25, opd
t_max_ (h)	4	24	1.2	3–5	4, 200 mg/12 h 3, 400 mg/24 h	4
C_max_ (µg/mL)	2	2.1	3.3	4.1	0.40, 200 mg/12 h 0.70, 400 mg/24 h	0.15
CL (L/h)	1.5, SD 3.3, MD		60.3, SD 7.79, MD	9.4	43.7	11.8
Vd (L/kg)	1.2		0.8–1	3.8	6.0	–a
F	>90%	75%	85%, SD^b^	40–45%	–a	–a
t_1/2_ (h)	45, SD 25–30, MD		2.4	52–76, SD 40–55, MD	30–40	47.7
Protein binding	62%		98%	>99%	99.9%	99.7%

t_max_: time to maximum plasma concentration (C_max_); CL: apparent plasma clearance; Vd: apparent volume of distribution; F: bioavailability; t_1/2_: half-life.

a Not available

b single-dose bioavailability of DLV tablets relative to an oral solution.

bid: twice daily; opd.: once daily; tid: three times daily. SD: single dose; MD: multiple doses.

### Nevirapine

#### Chemistry

NVP is a dipyridodiazepinone (11-cyclopropyl- 5, 11-dihydro-4-methyl-6H-dipyrido [3, 2-b: 2’, 3’-e] [1, 4]diazepin- 6-one). NVP is a low water soluble (0.1 mg/mL) and lipophilic drug (log *p*=3.89). Because of its weak basic character and ionization (pKa=2.8), NVP exhibits pH-dependent solubility [24]. At pH values below pKa, NVP is highly soluble in aqueous buffer. At higher pH values, NVP free-base solubility in water decreases asymptotically to approximately 0.1 mg/mL [25].

#### Biopharmaceutics and pharmacokinetics

The drug substance is classified as a Biopharmaceutics Classification System (BCS) Class II compound due to its low solubility and high intestinal permeability [26]. In fact, its oral bioavailability is higher than 90% [13].

Effective dosing regimen is 200 mg of NVP once daily for 14 days, followed by 200 mg twice daily. Following multiple doses, NVP peak concentrations appear to increase linearly in the dose range of 200–400 mg/day [14]. Data reported in the literature from 20 HIV-infected patients show steady-state C_max_ and C_min_ of 5.74 µg/mL and 3.73 µg/mL, respectively, and an area under the curve (AUC) of 109 µg.h/mL at a dose of 200 mg NVP twice daily [27].

NVP is widely distributed; it is highly lipophilic and is essentially non-ionized at physiologic pH. NVP readily crosses the placenta and has been found in breast milk. The CSF-plasma ratio for NVP is approximately 0.45 [28].

NVP is excreted mainly as glucuronidated metabolites in urine (80%) and 10% in feces. Less than 3% of an administered dose is excreted in urine as the parent compound [14].

Significant sex differences in plasma levels and pharmacokinetics of NVP have been reported. La Porte *et al*. [29] estimated that the median plasma NVP concentration was 22% higher in women than in men and the clearance 25% lower in women compared with men. Regazzi *et al*. [30] confirmed these differences with values of C_max_ 44% higher in women. On the other hand, NVP pharmacokinetics does not appear to change with age (range 18–68 years), and it has not been specifically investigated in patients over the age of 65 [16]. However, NVP concentrations are significantly associated with race, being 39% higher in black than in white patients [31].

#### Metabolism

In humans, NVP undergoes hepatic biotransformation to several hydroxylated metabolites (2-, 3-, 8- and 12-hydroxynevirapine) by CYP, and it is also an auto-inducer of isoenzymes 3A4 (CYP3A4) and 2B6 (CYP2B6). CYP3A4 was identified as the major enzyme involved in formation of 2- and 12-hydroxynevirapine, whereas CYP2B6 was the predominant enzyme forming 3- and 8-hydroxynevirapine [32].

#### Pharmacodynamics

NVP is a non-competitive inhibitor of HIV-1 RT, but it does not have a biologically significant inhibitory effect on HIV-2 RT or on eukaryotic DNA polymerases α, β, γ or δ [16].

HIV-1 isolates with reduced susceptibility (100–250-fold) to NVP emerge in cell culture. Genotypic analysis has shown mutations in the HIV-1 RT gene Y181C and/or V106A depending upon the virus strain and cell line employed. The most common resistance-associated mutation (RAM) *in vivo* is Y181C, but substitutions at positions 103, 106, 108, 181, 188 and 190 have been also observed [33, 34].

#### Safety and tolerability

NVP has been assigned to Pregnancy Category B by the FDA and one of its most relevant benefits is its efficacy in the prevention of mother-to-child transmission of the HIV-1 infection, with the drug being commonly prescribed to pregnant women and their children [35] Nevertheless, NVP is contraindicated in pregnant women with ≥250 CD4 cells/µL due to potential hepatic and cutaneous toxicity [36].

Severe and life-threatening skin reactions and hepatoxicity, including fatal cases as fulminant hepatitis, have occurred in patients treated with NVP. Skin reactions have included cases of Stevens-Johnson syndrome, toxic epidermal necrolysis and hypersensitivity reactions characterised by rash, constitutional findings and visceral involvement. The first 18 weeks of therapy with NVP are a critical period which requires close monitoring [35].

Sex-related differences in the toxicities of NVP have been reported. It has been found that adverse effects associated to NVP, such as rash and hepatotoxicity, are more common in women than in men [37, 38], and they have been related to the higher plasma levels in women than in men [39].

### Delavirdine

#### Chemistry

DLV belongs to a bisheteroarylpiperazines derivative (N-[2-({4-[3-(propan-2-ylamino) pyridin-2-yl] piperazin-1-yl} carbonyl)-1H-indol-5-yl] methanesulfonamide). It is a weak base (pKa=4.5) [40]; the aqueous solubility of DLV free base is 2.942 mg/mL at pH 1.0, 0.295 mg/mL at pH 2.0 and 0.810 · 10^−3^ mg/mL at pH 7.4 [41].

DLV is formulated as DLV mesylate, whose molar mass is 552.68 g/mol, and its log P is 2.98 [42].

#### Biopharmaceutics and pharmacokinetics

Oral bioavailability of DLV is 85% and is unaffected by food [43]. No data are available about BCS Class compounds.

DLV is manufactured as DLV mesylate (Rescriptor^®^), and the approved therapeutic dose is 400 mg every eight hours three times a day [43].

In a study with 13 HIV-1-infected patients, following administration of DLV mesylate (400 mg every 8 h, with meals), the systemic exposure (AUC) was 132±87 µM h and trough concentration (C_min_) was 11±9 µM [44].

The drug is highly protein bound (≈98%) in humans, predominantly to albumin [40], and has a low CNS penetration: cerebrospinal fluid concentrations are 0.39% of the corresponding plasma concentrations [45].

In healthy volunteers, similar amounts of radio-labelled drug are excreted in both feces (~44%) and urine (~51%) [40].

Data from population pharmacokinetics indicate that DLV plasma concentrations are 1.8 times higher in females than in males, although pharmacokinetic studies have not demonstrated a sex difference in metabolism [46]. Smith *et al*. [47] observed that women had a slightly lower clearance than men. No significant differences in DLV concentrations have been observed between different racial groups [43]. On the other hand, the pharmacokinetics of DLV has not been adequately studied in patients under 16 and over 65 years of age [43].

#### Metabolism

DLV is metabolized primarily by CYP3A4 into biologically inactive metabolites: N-dealkylated metabolite and unidentified pyridine hydroxy metabolites (including Met-7 and Met-7a) [48]. In contrast to the other first-generation NNRTIs (NVP and EFV), which are known to be enzyme inducers, DLV inhibits CYP3A4 [40]. It also inhibits *in vitro* CYP2C9, CYP2C19 and CYP2D6 [49].

DLV undergoes extensive hepatic metabolism, with <5% of the drug appearing unchanged in the urine in healthy volunteers. The major metabolic route is the CYP3A pathway, although ~20% is metabolized by CYP2D6.

#### Pharmacodynamics

DLV is highly selective for HIV-1 RT, and it has minimal effects against HIV-2 RT or human cellular DNA polymerase α or β [50]. Allosteric binding of the drug results in a stable conformational change in the polymerase site of the p66 subdomain of RT, converting it to an inactive state and restricting the flexibility of the p66 subunit domain. These changes, in turn, inhibit both the RNA- and DNA-directed DNA polymerase functions of the enzyme and consequently inhibit viral replication [21].

Resistance with DLV monotherapy develops rapidly. However, administration in combination with antiretroviral agents belonging to other classes markedly slows down the rate of acquisition of mutations [21]. The most frequent RAMs found in resistant viruses during treatment with DLV occur at codon positions 103 and/or 181, which confer resistance to nearly all NNRTIs. However, the potential for cross-resistance between DLV-resistant mutants and PIs or NRTIs is probably low [51].

#### Safety and tolerability

DLV has been assigned to Pregnancy Category C by the FDA and has been shown to be teratogenic in rats at doses producing exposure equal to or less than the expected human exposure at the recommended dose. There are no controlled data in human pregnancy. DLV should be used during pregnancy only when there are no alternatives and potential benefit outweighs potential risk to the foetus.

The most frequently reported drug-related adverse event among patients receiving DLV, either as monotherapy or in combination with NRTI(s), is severe skin rash, including rare cases of erythema multiforme and Stevens-Johnson syndrome. In most cases, the duration of the rash is less than two weeks and does not necessitate dose reduction or interruption of the therapy [43].

Others adverse effects occurring in at least 5% of adult patients in Phase I and II clinical trials evaluating DLV in combination with NRTIs are CNS side effects (anxiety, depressive symptoms and insomnia), respiratory system side effects (bronchitis, pharyngitis, sinusitis, upper respiratory infection and cough), digestive system side effects (nausea, vomiting and diarrhoea), asthenia or fatigue, headache, flu-like syndrome, localized pain, fever and generalized abdominal pain [43].

No sex- or age-related differences in the safety and effectiveness of DLV have been reported [43].

### Efavirenz

#### Chemistry

EFV is a benzoxazinone derivative ((S)-6-chloro-4-(cyclopropylethynyl)-1,4-dihydro-4-(trifluoromethyl)-2H-3,1-benzoxazin-2-one).

EFV is a crystalline nonhygroscopic lipophilic material, with a high log P (5.4). EFV is a weak acid, with a pKa of 10.2 [52]. It is practically insoluble in water, having aqueous solubility<10 µg/mL (pH 8.3) at 25°C. The aqueous solubility increased as the pH increased above 9, due to the loss of the proton on the amine of the carbamate [53].

#### Biopharmaceutics and pharmacokinetics

EFV is a Class II drug (low solubility and high permeability) according to the BCS, and its oral bioavailability is 40–45% [54].

Time-to-peak plasma concentrations (t_max_) are approximately 3–5 hours. In HIV-1-infected patients at steady state, mean C_max_, mean C_min_ and mean AUC are dose proportional following 200 mg, 400 mg and 600 mg daily doses. In 35 patients receiving 600 mg of EFV once daily, the steady-state C_max_ was 12.9±3.7 µM (mean±SD) and it was reached in 6–10 days, and the steady-state C_min_ was 5.6±3.2 µM [17].

Increases in C_max_ and AUC are dose proportional for 200, 400 and 600 mg EFV doses, but these increases are less than proportional for a 1600 mg EFV dose, suggesting reduced absorption at higher doses [55].

The drug is highly protein bound (>99%), and the CSF concentrations are 0.26 to 1.19% of the corresponding plasma concentrations [17].

Approximately 14–34% of a radiolabel dose of EFV 400 mg was excreted in the urine (less than 1% as unchanged drug) in the form of metabolites. and 16–61% was excreted in the feces as unchanged drug [17].

Burger *et al*. [56] estimated that the median plasma EFV concentration was 30% higher in women than in men. In one report, Barrett *et al*. [57] observed that the EFV clearance was slightly lower in females than in males (9.08 compared to 10.2 l/h in males). However, as the magnitude of this difference was small, the authors concluded that this finding does not suggest that EFV dose adjustments in men or women would be required.

Population analysis showed that black race resulted in changes in clearance that were, however, not clinically significant. These analyses showed that the Asian/Pacific Islander race is associated with a clearance reduction of 46%; therefore, an adequate statement has been included in the Summary of Product Characteristics (SPC) to reflect the potential higher exposure of EFV in this group of patients [17]. In another antiretroviral pharmacokinetic study, Stöhr *et al*. [31] reported that EFV serum concentration was significantly influenced by race (59% higher in black than in white patients; *p*<0.001). These differences have been explained by genetic variation, in particular in the polymorphic drug-metabolizing enzyme CYP2B6, with the 516G→T polymorphism occurring more frequently in black Africans than in white populations. The pharmacokinetic profile in children aged 3–16 years was evaluated in the study ACTG382. In this study, the pharmacokinetics of EFV appeared similar in children as in adults after correction for body size. No pharmacokinetic evaluations have been conducted in children weighing less than 13 kg or younger than three years or in patients older than the age of 65 [17].

#### Metabolism

*In vivo* and *in vitro* studies have demonstrated that EFV is metabolized in the liver predominantly by the CYP3A4 and CYP2B6 isoenzymes to inactive hydroxylated metabolites, including 8- and 7-hydroxy-efavirenz. CYP2B6 further catalyses the second step of hydroxylation of the 8-hydroxymetabolite to 8,14-dihydroxyefavirenz, and it is estimated that ~17% of 8-hydroxyefavirenz is oxidized to 8,14-dihydroxyefavirenz *in vitro* [58].

EFV has been shown to induce CYP enzymes, with the induction of its own metabolism. Doses of 200–400 mg per day for 10 days resulted in a lower than predicted extent of accumulation (22–42% lower) and a shorter terminal half-life of 40–55 hours (single dose half-life 52–76 hours) [17].

#### Pharmacodynamics

EFV is a non-competitive inhibitor of HIV-1 RT, but it does not have a significant inhibitory effect on HIV-2 RT or on human cellular DNA polymerases α, β, γ or δ [17].

The primary mechanism of viral resistance or reduced susceptibility to EFV appears to be the mutation of HIV RT, although this has not been fully determined. Like the other NNRTIs, EFV has a low genetic barrier to resistance and selects mutations that usually involve the regions of HIV RT. The K103N mutation is the most characteristic EFV RAM [59]. Other mutations affect amino acids at positions 100, 106, 108, 181, 188, 190 and 225 [33].

Several studies that compared EFV-based regimens with other regimens have shown that treatments containing EFV and two NRTIs were associated with a better virologic response than some PI-based regimens and triple-NRTI-based regimens [60].

EFV must not be used as a single anti-HIV agent, as a resistant virus emerges rapidly when EFV is administered as monotherapy. The choice of new antiretroviral agents to be used in combination with EFV should take into consideration the potential for viral cross-resistance. Cross-resistance between EFV and NRTIs is considered low because the drugs bind at different sites and have different mechanisms of action.

Cross-resistance between EFV and PIs is unlikely as the enzyme targets involved are different.

#### Safety and tolerability

EFV is FDA Pregnancy Category D, and the 2009 revision of the World Health Organization (WHO) guidelines does not recommend its use during the first trimester of pregnancy or in women of childbearing age in the absence of an effective contraceptive method due to the concern for teratogenicity. In addition, the 2009 revision of the WHO guidelines states that if a woman already on EFV is diagnosed as pregnant 28 days before gestation, EFV should be stopped and substituted by NVP or a PI. If a woman is diagnosed as pregnant after 28 days of gestation, EFV should be continued. Finally, there is no indication for abortion in women exposed to EFV in the first trimester of pregnancy [61].

Significant elevation of liver enzymes and hepatic failure can occur with or without co-infection with hepatitis B or C [17].

EFV has a high rate of CNS side effects (up to 55%), including headache, dizziness, insomnia, impaired concentration, agitation, amnesia, somnolence, abnormal dreams, fatigue and hallucinations [17]. CNS toxicity has been reported more frequently in adult and paediatric patients with EFV trough plasma concentrations >4 µg/mL [62]. Occasional post-marketing reports of death by suicide or psychosis-like behaviour in patients taking EFV have been made [17].

Other commonly reported adverse effects include skin rashes, which usually appear as mild or moderate maculopapular eruptions; these occur within the first two weeks of therapy and in most patients resolve within a month with continuing EFV administration. Children have a higher incidence of rash (46% of children compared to 26% of adults) [63]. Erythema multiforme and Stevens-Johnson syndrome are extremely rare in adults (0.1%) and more frequent in children (5%), and require discontinuation of EFV.

Gastrointestinal effects (nausea, diarrhoea, vomiting, dyspepsia, anorexia and malabsorption) have been reported in up to 14% of adults receiving EFV [17].

While patients on EFV-based ART show fewer atherogenic lipid changes than patients treated with the PI-based regimen, EFV may increase the concentration of total cholesterol and triglycerides [64]. Lipodystrophy, moderate or severe pain, abnormal vision, arthralgia, asthenia, dyspnoea, gynecomastia, myalgia, myopathy and tinnitus have also been reported [17].

Sex-related differences in the toxicities of EFV have been reported. Female sex was a strong independent risk factor for developing rash, as confirmed by Tran *et al*. [65]. Factors involved in the predisposition of women to this side effect are unclear and warrant further exploration. The role of steroid hormones, oral contraceptives, menstruation and pregnancy, as well as the potential role of sex-related differences in the CYP system, given that EFV is metabolized via this route, should be analysed [66]. However, black race was not a risk factor for the development of rash [67].

## Second-generation NNRTIs

Second-generation NNRTIs currently include ETR and RPV, which belong to the family of di-aryl-pyrimidine (DAPY) compounds. These antiretroviral drugs display a better resistance profile and an increased genetic barrier to the development of resistance. Additionally, second-generation NNRTIs have a convenient dosing schedule, with potential for co-formulation with other antiretroviral drugs [68]. Table 3, [13–23] shows the pharmacokinetic characteristics of these NNRTIs.

ETR is used in treatment-experienced multidrug-resistant HIV-infected adult patients [69], and RPV has been tested in treatment-naïve patients and shows comparable efficacy with EFV [70].

### Etravirine

#### Chemistry

ETR belongs to the family of DAPY compounds, and its chemical name is 4-[6-amino-5-bromo-2-(4-cyanoanilino)pyrimidin-4-yl]oxy-3,5-dimethylbenzonitrile.

ETR is practically insoluble in water (0.01 mg/mL) over a wide pH range; however, it is soluble in propylene glycol and ethanol. The drug is highly lipophilic with a log P of 5.2 and a pKa of 3.5 [71].

#### Biopharmaceutics and pharmacokinetics

ETR has low solubility and permeability and is categorized as a BCS Class IV compound [72].

Due to its high lipid solubility, ETR produces bioavailability issues. For this reason, and to increase the solubility and the gastric residence time, ETR should be taken with food [73]. After oral administration with food, absorption is increased by 50% and its maximum plasma concentration is generally achieved within four hours [74].

ETR is available in 100 mg tablets, and its approved dosage for clinical use is of 200 mg twice daily [75].

The drug is highly protein bound (99.9%), primarily to albumin (99.6%) and alpha 1-acid glycoprotein (97.66 to 99.02%) *in vitro*, and its distribution to compartments other than plasma (e.g., cerebrospinal fluid or genital tract secretions) has not been evaluated [23].

Elimination occurs primarily in faeces (81.2 to 86.4% of the administered dose) and bile (11%), with a negligible (1%) amount detected in the urine [76].

Few data are available concerning races other than Caucasian. A difference in treatment response was observed in Latin America, the United States and Canada compared to the European Union (with a trend for lower response in the EU population) [74]. Furthermore, sex and age do not affect the pharmacokinetics of ETR [77].

#### Metabolism

*In vitro* experiments with human liver microsomes (HLMs) indicate that drug metabolism is primarily performed by several CYP isozymes (CYP3A, CYP2C9 and CYP2C19). The most common metabolic pathway is methyl hydroxylation, followed by glucuronidation of the metabolites; aromatic hydroxylation represents a minor pathway. Metabolites formed by methyl hydroxylation are at least 90% less active than ETR against wild-type HIV in cell cultures and are found mainly in urine [76].

#### Pharmacodynamics

ETR is an NNRTI, designed to be active against HIV-1 that presents resistance to EFV and NVP, by K103N and Y181C mutations, respectively [78]. Given its activity against viruses with mutations, ETR may be an effective option for patients with limited or no available treatment choices [69]. Its mechanism of action consists in binding directly to RT in multiple conformations, allowing a more robust interaction with the enzyme, even in the presence of mutations [79] and it blocks RNA-dependent and DNA-dependent DNA polymerase activities by causing a disruption of the enzyme's catalytic site. [74].

Resistance to ETR has been reported, but it is uncommon in patients who have not received ETR before [78].

Resistance development is dependent on the presence of multiple coexisting mutations, demonstrating the high genetic barrier of ETR. RAMs of ETR have been characterized both *in vitro* and *in vivo*, and these include known and also novel NNRTI mutations [80].

ETR is considered a second-generation NNRTI because of its limited cross-resistance caused by its structural differences when compared to other NNRTIs.

#### Safety and tolerability

ETR is an FDA Pregnancy Category B drug, and it is not recommended while breastfeeding [81]. No data are available about safety and efficacy of ETR in paediatric patients [22].

The primary adverse effects observed in the DUET studies have been rash and gastrointestinal effects, primarily nausea [82, 83]. Women experienced rash more frequently than men (30% vs. 18%). A higher frequency of hepatotoxicity or CNS adverse effects in patients receiving ETR compared with those receiving placebo has not been found [82, 83].

Stevens-Johnson syndrome and other severe life-threatening reactions occurred in <0.1% of all ETR recipients (a frequency lower than that reported with EFV) [84].

In a study that enrolled treatment-experienced adults aged 18 years or older, Hodder *et al*. [85] reported that women experienced more nausea (24.4% vs. 11.4%) and rash-related events (21.0% vs. 15.9%), but diarrhoea to a lesser extent (15.1% vs. 21.6%), compared with men. Grade 3–4 hypertriglyceridemia was more common in men (9.3%) than women (1.1%). Treatment discontinuation was slightly higher in women than in men (9.2% and 8.0%, respectively) due to adverse events.

Race did not appear to substantially affect ETR exposure [77], and the safety profile in patients above 65 years old has not been well established [74].

### Rilpivirine

#### Chemistry

RPV (4-[[4-[4-[(E)-2-cyanoethenyl]-2,6-dimethylanilino]pyrimidin-2-yl]amino]benzonitrile) is a very poorly water-soluble (<0.1 mg/mL) and oil-soluble diarylpyrimidine derivative [86]. RPV is formulated as RPV hydrochloride whose molar mass is 402.88 g/mol. Its pKa is 5.6 and log P between 1-octanol, and a phosphate solution (pH 7) is 4.86 [19].

#### Biopharmaceutics and pharmacokinetics

RPV is a drug with high oral bioavailability despite its high hydrophobicity. The substance is classified as a BCS Class II compound (low solubility and high permeability) [87].

RPV is manufactured as Edurant^®^ in tablets containing RPV hydrochloride 27.5 mg (equivalent of 25 mg of RPV). Effective dosing regimen is 25 mg tablet of RPV once daily. The solubility and systemic absorption are pH dependent, as demonstrated by an increased bioavailability in an acidic environment [88]. RPV should be administered with food, as under fasting conditions, C_max_ and AUC decrease by 46% and 43%, respectively. Moreover, both parameters are reduced by 50% when RPV is given with a protein-rich nutritional drink [89].

RPV is 99.7% bound to plasma proteins, mainly to albumin, but its distribution outside plasma is unknown [19].

Only trace amounts of unchanged RPV (<1% of dose) are detected in urine, whereas 25% of the administered dose has been found in faeces [90].

There is a statistically significant effect of sex on apparent oral clearance (CL/F) of RPV, with CL/F 13.6% lower in females than in males [91]. Data on RPV in elderly patients are rather scarce, and hence no conclusions regarding the elderly can be made [92].

#### Metabolism

RPV is primarily metabolized by the CYP 3A isoenzyme system. Therefore, caution should be taken when administering inhibitor or inducer drugs, which may lead to increased or decreased concentrations of RPV, thereby increasing the risk of adverse effects or promoting virologic failure or resistance to RPV [86].

#### Pharmacodynamics

RPV activity is mediated by non-competitive inhibition of HIV-1 RT. RPV does not inhibit the human cellular DNA polymerases α, β and γ. RPV has a high genetic barrier to resistance development [93].

Sensitivity to RPV is not affected by the presence of most single NNRTI RAMs, including those at positions 100, 103, 106, 138, 179, 188, 190, 221, 230 and 236 [93].

#### Safety and tolerability

Safety in pregnancy has not been directly assessed in pregnant women as they are excluded from clinical trials; however, RPV has been classified as a pregnancy class B [88].

The most extensive safety and tolerability data to date come from the 96-week Phase IIb randomized trial, in which the median duration of follow-up extended over 100 weeks. It was proven that RPV was well tolerated with lower incidences of neurological and psychiatric adverse events, rash and lower lipid elevations than those with EFV [94].

Two Phase III comparative trials (ECHO and THRIVE) were developed to demonstrate the efficacy and safety of RPV versus EFV. When the analysis ended after 48 weeks, RPV 25 mg once daily and EFV 600 mg once daily had comparable response rates, but RPV displayed a better tolerance than EFV. RPV gave smaller incidences of adverse events leading to discontinuation, treatment-related grade 2–4 adverse events, rash, dizziness, abnormal dreams and nightmares and grade 2–4 lipid abnormalities [95]. Women had significantly higher nausea rates than men (19% vs. 11.2%, respectively). In contrast, psychiatric adverse events, such as abnormal dreams and nightmares, were significantly more frequent in men (4.1% vs. 11.4%, respectively) [96].

## Next-generation NNRTIs

Currently, the next generation of NNRTIs is undergoing clinical development. Information about ongoing clinical research studies is shown in Table 4, [97–104]. These drugs have not been approved by the FDA or European Union for use against HIV. NNRTIs in development include lersivirine (UK-453,061), GSK 2248761 (formerly IDX 899), RDEA806, BILR 355 BS, (+)-Calanolide A, MK-4965, MK-1439 and MK-6186. Lersivirine and GSK 2248761 are the most advanced compounds being investigated (Phase IIb). However, in February 2011, the FDA placed GSK 2248761 trials on clinical hold, and in February 2013 the company responsible for the development of lersivirine decided to halt the development programme.

**Table 4:** Current status of clinical trials of next-generation NNRTIs [97–104]

Research code	Generic name	Sponsor	Status
UK-453,061	Lersivirine	ViiV Healthcare	Phase IIb
GSK 2248761 (formerly IDX 899)	–	ViiV Healthcare	Phase IIb
RDEA806	–	Ardea Bioscience	Phase IIa
BILR 355 BS	–	Boehringer Ingelheim Pharmaceuticals	Phase IIa
(+)-Calanolide A	–	Sarawak MediChem Pharmaceuticals	Phase I
MK-4965	–	Merck Research Laboratories	Phase I
MK-6186	–	Merck Research Laboratories	Phase I
MK-1439	–	Merck Research Laboratories	Phase I

To our knowledge, development of atevirdine, capravirine, dapivirine (TMC120), DPC083, emivirine, GW5634, GW678248, loviride, HBY-097 and PNU142721 has been suspended [105].

### NNRTIs under Phase II trials

Lersivirine, GSK 2248761, RDEA806 and BILR 355 BS are undergoing Phase II trials. They have been demonstrated to be safe, well tolerated and potent in antiretroviral activity in short-term monotherapy studies with antiretroviral-naïve HIV-infected subjects. These newer NNRTIs retain a marked activity against wild-type and a broad range of HIV-1 strains, including NNRTI-resistant mutants with single mutations (K103N or Y181C) and double mutations.

#### Lersivirine

Lersivirine (UK-453,061) is a next-generation NNRTI belonging to the pyrazole family. It exhibits pH-dependent solubility; its solubility at acidic pH (<2) is approximately threefold greater than that in the remainder of the physiologically relevant pH range (pH 2 to 7) [106].

Lersivirine has a unique binding interaction within the RT binding pocket. It is active against wild-type HIV-1 and a broad range of drug-resistant viral strains [107].

Lersivirine is metabolized by glucuronidation via UGT2B7 and by CYP3A4-mediated oxidation [108].

Moreover, the safety and tolerability of lersivirine have been studied in both HIV-infected and healthy subjects with favourable results [109]. A Phase IIb trial reported non-inferiority to EFV in treatment-naïve patients [110]. Nevertheless, in February 2013, ViiV Healthcare, the company responsible for lersivirine development, decided to bring the development programme to a stop as lersivirine did not provide a clear improvement over existing medicines in the NNRTI class [111].

#### GSK 2248761

GSK 2248761 (formerly IDX 899) is an aryl phosphinate-indole with a 50% effective concentration (EC_50_) of 36.9 ng/mL and activity against EFV-resistant strains [98]. GSK 2248761 is a weak CYP3A4 and CYP2D6 inhibitor [112]. In a clinical study designed to evaluate potential interactions of this drug with antiretroviral therapies or supportive therapies used in patients with HIV-1 infection, decreased plasma levels of lopinavir and increased plasma levels of simvastatin were observed when these drugs were administered with GSK 2248761 [113]. In February 2011, ViiV Healthcare announced that the FDA had placed a hold on its development, due to four reports of seizures as part of a clinical trial involving treatment-experienced patients, and it is unclear if or when development will continue [114].

#### RDEA806

RDEA806 belongs to the family of triazoles. It has an EC_50_ of 1.7 ng/mL and similar activity in the presence of K103N and other common single mutations in the NNRTI binding region [115].

#### BILR 355 BS

BILR 355 BS is a dipyridodiazepinone compound that requires ritonavir as a booster [116]. The *in vitro* data show that the EC_50_ against wild-type HIV-1 is 0.26 ng/mL, while the EC_50_ against common NNRTI-resistant viruses ranges from 1.5 to 13 ng/mL. *In vitro* data indicate that BILR 355 BS exhibits a low level of cross-resistance in a broad spectrum of viruses that are highly resistant to NVP, EFV and DLV [116]. Lopinavir and ritonavir decrease exposure to BILR 355 BS [117]. Similarly, concomitant administration of ritonavir-boosted BILR 355 BS with lamivudine/zidovudine [118] and emtricitabine/tenofovir disoproxil fumarate [119] led to a modest decrease in exposure to BILR 355 BS and an increase in exposure to other compounds.

### NNRTIs under Phase I trials

(+)-Calanolide A, MK-4965, MK-1439 and MK-6186 are in Phase I trials to evaluate their safety, tolerability and favourable pharmacokinetic profile.

#### (+)-Calanolide A

(+)-Calanolide A was first isolated from a tropical tree (*Calophyllum lanigerum*) in Malaysia [120]. The activity (i.e., the 50% effective concentration) of the compound ranged from 0.02 to 0.5 mM [101].

#### MK-4965 and MK-6186

MK-4965 and MK-6186 have been recently discovered and developed by Merck. This novel class of next-generation NNRTIs contains the pyrazolo[3,4-b]pyridine fragment and the pendant biaryl ether moiety [121, 122]. Both of them display excellent activities against the wild-type virus and the two most prevalent NNRTI-resistant RT mutants (K103N and Y181C). MK-4965 is an NNRTI containing biaryl ether and indazole moieties. The compound retained an EC_95_ of 4.4 nM against the wild-type virus and excellent activities against viruses with the K103N, Y181C and K103N/Y181C mutations (an EC_95_ of 13, 30 and 128 nM, respectively) [123]. MK-6186 is a novel NNRTI containing a chlorobenzonitrile and two indazole rings. The EC_95_ of MK-6186 was 13, 16, 60 and 109 nM against the wild-type virus and the K103N, Y181C and K103N/Y181C viruses, respectively [124].

#### MK-1439

MK-1439 shows excellent potency in suppressing wild-type virus replication with an EC_95_ of 20 nM as well as K103N, Y181C and K103N/Y181C mutant viruses with an EC_95_ of 43, 27 and 55 nM, respectively [125]. Recently, Merck has announced a study to compare MK-1439 to EFV, both combined with tenofovir/emtricitabine (the drugs in Truvada^®^), in treatment-naïve individuals.

## Conclusions

In conclusion, NNRTIs have been classified into three groups depending on their approval by the European Union and/or the FDA and their genetic barrier to the development of resistance.

First-generation NNRTIs, NVP, DLV and EFV, are drugs with a low genetic barrier that require only a single mutation to confer resistance, and cross-resistance is common. In contrast, second-generation NNRTIs, ETR and RPV, are compounds with a higher genetic barrier. Both generations have been approved by the FDA and also, with the exception of DLV, by the European Union. Clinical development of some NNRTIs is currently ongoing, and these drugs have been classified as next-generation NNRTIs. As second-generation NNRTIs, these compounds have the advantage of being drugs with activity against HIV-1 wild-type and clinically relevant mutant strains.
